# Annotations

**Published:** 1896-11-07

**Authors:** 


### ANNOTATIONS.

### Flowers in Bedrooms.

An American actress, it is announced, has recently escaped death only by a hairsbreadth in consequence of the too oppressive odour of violets in her bedroom. An enthusiastic admirer having sent her a large hamper full of violets, she distributed the flowers in vases about her bedroom, and, after shutting down her window, went to sleep. Next morning she was discovered in a collapsed condition, and was with the greatest difficulty brought back to life. This is an American story, and may perhaps be questioned by the sceptical. But the present writer well remembers a similar experience which was related to him by the late Sir Andrew Clarke, not long before his death. Sir Andrew was called to a patient who had been suffering some days with obscure internal symptoms, of which the family doctor had failed to find the cause. The great physician examined the patient in turn, and could not satisfy himself as to the exact nature or cause of the attacks. He, however, prescribed, and hoped for the best. A few days afterwards he was called again, the patient being manifestly in a very dangerous condition; and, again, after making the most thorough investigation of the patient and attendant circumstances, failed to satisfy his judgment as to the true nature or the cause of the illness. Being thus brought to a dead stand, Sir Andrew looked round the room to see if he could find evidence of the presence of damp or mould. All the walls were dry, and the place seemed in every way healthy. Proceeding to investigate further, he found, hidden away out of sight, a vase of drooping flowers—hyacinths, if memory serves correctly—several days old. The water in the vase, or what was left of it, was of a very offensive odour. Thereupon a change of room was ordered for the patient, with the result that recovery commenced at once, and was complete within a very few days. It is not necessary to comment at length upon cases like these. They tell their own story, and point their own moral. The rule should be that, where flowers are kept in bedrooms, they should be changed frequently, and those which yield a heavy odour should not be preserved after the day is over. In sitting-rooms the case is somewhat different; but even in them flowers should not be kept for more than a few days, and the vases in which they are placed should be well washed out with hot water once or twice a week.

### Giants and Dwarfs.

The paper read by Mr. Hastings Gilford at the last meeting of the Royal Medical and Chirurgical Society raised some very interesting questions as to the relations between dwarfism and giganticism. Of course every one admits that some men may be large and others small without in any way departing from the normal in regard to the relation of their different parts, and that we may thus have men who are perfect though gigantic in every part, while also we may have dwarfs who are but men on a tiny scale. But it is pointed out that neither all giants nor all dwarfs are built with such symmetry, and that while tiny dwarfs may have big heads and an intelligence quite precocious, giants are very commonly not built on an equally large scale all through. The idea is then suggested that both dwarfism and giganticism are but diverse manifestations of one condition—disease if one likes so to call it—the dominant feature of which is not largeness nor smallness, but lack of proportion between the different parts, taking different forms according to the time of life when it occurs. Under the name of acromegaly, we know of this as a disease which shows itself as an abnormally large development of the extremities, and it is said that many so-called giants are but specimens of this disease, and that some of them are as small in some parts as they are large in others. On the other hand, in certain cases which were described by Mr. Gilford, while the frame as a whole was small, the head was large, as also were certain parts of the skeleton; and the intellectual development, although not perhaps marked by brilliancy, was at least far more advanced than that of other children of the same age. The possibility of such disturbances of proportion being due to some such morbid condition affecting the development as to deserve the name of a disease is all the more interesting from the fact that, although such cases as those related by Mr. Gilford are undoubtedly rare, no one can walk about in that vast pathological museum which the streets of London form to those who have an observant eye, without perceiving that in slighter degree signs of partial dwarfism or giganticism are by no means of uncommon occurrence among people who, in one way or another, succeed in earning their living in competition with normal man—if there be such an animal.

### A New Danger for London.

The Abney Park Cemetery Company, who are making great efforts to establish a new cemetery on the west side of and very near to Hampstead Heath, are, we are glad to see, meeting with strenuous resistance in the neighbourhood. Of course, London must bury its dead, at any rate until the more rational method of cremation comes into general favour; and equally of course it must have cemeteries for the purpose. But in determining the location of such cemeteries the metropolitan authorities, acting on behalf of all the living inhabitants of the metropolis, must insist upon the fulfilment of at least two indispensable conditions. The first of these conditions is that any new cemetery which may be proposed shall be located at a sufficient distance from the living population; and the second is that the soil of which the cemetery is composed shall be suitable for purposes of extensive burial. The proposed cemetery at Hampstead fails entirely to fulfil either of these conditions; and that being so, the proposal of the Abney Park Company should be firmly and absolutely negatived at once, and without any further consideration. For let any man of plain sense consider the two conditions named in their relation to the actual facts of the case. A cemetery, we have said, should be located at a sufficient distance from the living. The proposed new Abney Park Cemetery is within one-third of a mile of the metropolitan area. It requires nothing but the most elementary knowledge of matters sanitary to enable any man to see that such a distance is absurd for a new cemetery. In the second place, the soil suitable for burials is a light, porous, dry soil; and this kind of soil is indispensable. The soil of the proposed new cemetery is a heavy clay. Of course, there are other objections urged. But these two are fatal. Either of them, indeed, is fatal by itself. Let the authorities and the public, therefore, keep these two conditions in mind, and they will be furnished with the amplest of sanitary reasons for refusing the petition of the Abney Park promoters of a large area of insanitation for North-west London.

